# Cersten Jakob: Von Prüfungsangst zu Prüfungsmut, von Lampenfieber zu Auftrittslust

**DOI:** 10.3205/zma000969

**Published:** 2015-08-17

**Authors:** Angelika Kursch

**Affiliations:** 1 Medizinische Hochschule Hannover, Studiendekanat Medizin, SkillsLab, Kommunikationskurse, Hannover, Deutschland

## Recension

1. The author of this book wants to reveal ways leading to more confidence and calmness in exams and while performing in front of small or larger audiences. A short introduction is followed by a definition of the term stage fright and a distinction between useful and obstructive types (stage fright). The complexity of the phenomena stage fright and exam anxiety is vividly visualised on the basis of a mind map. The following chapter with the heading “What to do” and “How to address which issues?” lead to first action steps. Worksheets provide a substructure for the first evaluation of the obstructive symptoms. The following chapter deals with the scope of action that occurs while working on symptoms and causes of stage fright and exam anxiety. The author uses examples to illustrate the connection between nonverbal communication, the sound of the voice, speaking pattern and attitude, followed by stimulations and tutorials about body language and voice improvement. Two chapters address to mental preparation and the resulting solution approaches, as well as to the influencing factors. Chapter 1 deals with the enhancement of self-acceptance and the author’s distinction between “perfect” and “optimal”. For this purpose the chapter introduces helpful relaxation techniques. In Chapter 2, the author focuses on the causes of stage fright and exam anxiety. He introduces methods of goal attainment, activation of resources, self-negotiation and their specific effects. These techniques of the NLP (neurolinguistic programming), the Wingwave-Coaching, auditive kinaesthetic Intervention, a variation of knocking techniques and kinesiological exercises belong to these methods of goal achievement. Additionally, the book gives a substantial overview of helpful coaching- and therapeutic methods, customized for these challenging occasions. In addition to this, the book address a adapted diet and the usage of endogenous drugs. Chemical stimulants and their effects are mentioned and discussed controversially. The book ends with elaborated checklists, which can be helpful to prepare presentations and exams successfully.

2)… and another book concerning the topic of stage fright, performance and exams anxiety. A range of guides can be found.

Viewing the phenomenon of stage fright from different angles and to propose reconciliation with useful stage fright, seems to be a sensible approach. The author demonstrates convincingly how the outer, observable features such as mimic, gesture, voice and posture influences the inner posture respectively inner doctrines…and also vice versa by mental preparation techniques. On the basis of citations, examples and own experience the author, as a trained actor and studied linguist, succeeds to render the complex and delicate topic stage fright and exam anxiety accessible to the reader. However, a lot of the citations used in examples might miss their mark with the younger generations since the cited persons stem from a previous generation. Additionally the book contains two parts with anecdotes concerning women, which seem a bit old-fashioned. 

The contents between the chapters mental preparation 1 and 2 are adaptable and therefore useful. Here one can find useful suggestions to structure different presentation formats, to handle disturbances, possible technical breakdowns and blackouts during a presentation confidently. The extensive view on the complexity of stage fright and exam anxiety is one of the strengths of this book. This can especially be found in the two chapters on mental preparation. This part warrants careful and thorough reading, because it lists an extensive selection of academically and experientially proven methods for all those who are interested. Those are useful application methods of short and long term positive influence. 

The book by Cersten Jacobs seems right for those, who are willing to make a decision concerning their professional challenges. Examinees, teachers or individuals undergoing training who want to start acting. It`s for those who are determined to address their issues and provides them with the necessary tools to achieve their aim. The required tools are described extensively and comprehensively. 

## Competing interests

The author declares that she has no competing interests.

